# Examining the Structure of Directed Motivational Currents (DMCs) Among Secondary and Tertiary English as a Second Language Learners

**DOI:** 10.3390/bs15081066

**Published:** 2025-08-06

**Authors:** Chuanwei Huo, Lawrence Jun Zhang, Jason M. Stephens

**Affiliations:** Faculty of Arts and Education, University of Auckland, 10 Symonds St., Auckland 1010, New Zealandjm.stephens@auckland.ac.nz (J.M.S.)

**Keywords:** L2 motivation, Directed Motivational Currents (DMCs), DMC triggers, flow theory, Expectancy-Value Theory (EVT)

## Abstract

Motivation remains a central concern in second language (L2) and English as a foreign language (EFL) education, yet its underlying mechanisms are insufficiently understood. This study employs the theory of Directed Motivational Currents (DMCs) to explore periods of intense, sustained L2 motivation among Chinese adolescent EFL learners across secondary and tertiary levels. Through in-depth interviews with ten participants, this research identified the conditions (e.g., collaborative peer dynamics, vivid goal visualization) that triggered their DMC experiences. The data also highlighted how facilitative elements—such as clear starting points, personalized goal alignment, behavioral routines, and timely feedback—played a crucial role in initiating and sustaining these motivational currents. These findings contribute to DMC theory by revealing how intrinsic and extrinsic factors jointly foster and maintain high levels of motivation over time, offering valuable insights for designing targeted interventions to enhance EFL motivation and learning among Chinese adolescents.

## 1. Introduction

Aligned with the motivational dynamic shift in second language acquisition (SLA) (Dörnyei et al., 2015; Dörnyei & Ushioda, 2011), numerous theoretical and empirical studies have examined motivational experiences during second language (L2) learning (Ibrahim, 2016a, 2016b; Muir, 2016; Shen et al., 2025; Oh & Kim, 2024). Dörnyei and his colleagues (Dörnyei et al., 2016) introduced Directed Motivational Currents (DMCs) to address the shortcomings of previous research by emphasizing the complexity, dynamism, and process-oriented nature of motivation (Chang & Zhou, 2023; Xodabande & Babaii, 2021; Zarrinabadi & Tavakoli, 2017). At the same time, extensive studies underscored the significant influence of DMCs on motivational and cognitive processes, well-being, and achievement in L2 learning (Pietluch, 2018; Safdari & Maftoo, 2017; Shen et al., 2025). However, consensus concerning the mechanism underlying DMCs remains elusive (Gümüş & Muir, 2020).

A DMC has been defined as a long period of powerful energy directed by a clear goal/vision and supported by enduring momentums produced by the accomplishment of sub-goals (Dörnyei et al., 2014; Henry et al., 2015). Dörnyei and his colleagues (Dörnyei et al., 2016) also posited that a DMC includes a more immovable determination than ordinary motivational forces. Thus, students experiencing a DMC not only demonstrate the high motivation typically exhibited by outstanding students, but they also embody an extraordinarily level of goal commitment and task perseverance. Dörnyei, Henry, & Muir (Dörnyei et al., 2015) claimed that when individuals in a DMC experience they are driven by a clear vision, enabling them to navigate challenges without succumbing to negative affect, thereby sustaining effort through repetitive or tedious tasks such as vocabulary memorization (Ibrahim, 2016a; Zarrinabadi & Khodarahmi, 2023).

Despite a growing body of literature, critical gaps persist in our understanding of this complex motivational construct. One gap is that little attention has been paid to the specific triggering mechanism of DMCs. Few studies to date have explored the psychological and situational factors that contribute to the initiation of DMCs. A second critical gap in DMC research concerns the factors associated with the maintenance of intense motivational surges (i.e., the mechanisms for re-triggering and sustaining motivation, particularly in overcoming distractions or competing impulses that derail vision-oriented goals). Additionally, previous studies have typically examined DMCs within one educational stage but have not compared them across the secondary and tertiary levels. DMCs are inherently intense and complex motivational phenomena whose situational nature demands rigorous comparative analyses across educational contexts and demographic groups to elucidate how these factors jointly shape motivational trajectories. Such analyses may also uncover previously undefined features, thereby reinforcing their role as a nuanced framework for understanding motivation as a dynamic, contextually embedded process.

## 2. Literature Review

### 2.1. Key Components of Directed Motivational Currents (DMCs)

The DMC construct has three main elements: “vision-oriented, prominent identifiable structure, and supportive and positive feelings” (Henry et al., 2015, p. 330). Vision-oriented refers to an individual’s ideal self, or the “personalized goal that the learner has made his/her own by adding to it the imagined reality of the goal experience” (Dörnyei & Chan, 2013, pp. 454–455). As they suggest, a DMC has a directional nature—it is fueled by a highly valued goal, which assist the individual to apply energy for goal achievement (Dörnyei et al., 2016; Muir & Dörnyei, 2013).

The second prominent element of a DMC is an *identifiable structure*. Specifically, a DMC has three central features for the structured and tailored pathway, the first one being a clearly legible *starting point*. A DMC should be intentionally and clearly activated by something specific (Dörnyei et al., 2015). This refers to a sudden trigger which is consciously and explicitly pulled. Once this occurred, the ‘current’ of the DMC initiates and drives the subject into the second feature of the presence of *regular progress checks*. During the process of achieving an ultimate goal, there are usually several sub-goals that serve as staged targets and ‘as criteria to evaluate and confirm progress’ (Dörnyei et al., 2016, p. 100). The third feature is that *learning routines* or practices may be developed while in a DMC (Dörnyei et al., 2014; Muir & Dörnyei, 2013). In a 2016 study (Ibrahim, 2016a), a 29-year-old male learner reported that he developed the routine of studying late at night as a result of intense, highly focused energy he experienced at this time. This phenomenon is not rare as evidence in subsequent studies has supported the idea that L2 learning behaviors in DMCs tend to become habitual routines (Ghanizadeh & Jahedizadeh, 2017; Safdari & Maftoo, 2017; Gümüş, 2019; Chang & Zhang, 2020). This structure does not “merely frame the process, but also plays a vital role in facilitating the unfolding action” (Dörnyei et al., 2014, p. 14).

Positive emotionality is the third component of DMCs, where a personalized goal becomes central to one’s identity and fuels motivation. Henry et al. (2015) describe this state as actualizing potential and generating a profound sense of pleasure and satisfaction. This phenomenon is closely linked to eudaimonic well-being, which encompasses feelings of rightness and fulfillment in one’s actions and self-concept (Waterman, 2008). In the context of DMCs, routine tasks that might ordinarily be perceived as mundane are transformed into meaningful, joyful steps toward a higher purpose (Dörnyei et al., 2015). Unlike the intrinsic rewards one experiences in a state of “flow” (Csikszentmihalyi, 1988), positive emotionality in DMCs emerges from making meaningful progress toward a significant future vision, thereby driving sustained achievement and personal development.

Moreover, DMCs are inherently situational: They arise from a clear trigger within a learning ecology and then cascade into sustained, high-energy engagement punctuated by sub-goals and progress checks (Dörnyei & Muir, 2019). An ecological perspective reminds us that learners are embedded in overlapping social, cultural, and instructional contexts that both shape and sustain these currents (Consoli, 2024). Pedagogically, process-oriented, project-based designs that mirror authentic L2 use, by scaffolding tasks, feedback, and group dynamics, can deliberately activate DMCs in the classroom (Muir, 2020). Such context-sensitive applications underscore DMC’s transformative promise for enhancing L2 education and motivation research.

### 2.2. Flow Theory

Flow Theory, introduced by Csikszentmihalyi (1975), describes a state of optimal experience characterized by complete absorption in an activity, intrinsic enjoyment, and the temporary loss of self-consciousness. While both Flow and DMCs involve deep focus and immersion, they differ in several critical aspects (Dörnyei & Ryan, 2015). Flow is typically associated with short-term, intrinsically rewarding tasks, whereas DMCs are sustained motivational states geared toward achieving long-term, goal-directed outcomes. In DMCs, positive emotionality is derived not merely from task enjoyment but from the incremental satisfaction of moving closer to an overarching objective (Dörnyei et al., 2015). Moreover, DMCs often extend over prolonged periods—a reflection of the inherently time-consuming nature of language learning—whereas Flow episodes generally last only hours or days (Henry et al., 2015).

### 2.3. Goal-Setting Theory and Expectancy-Value Theory

Goal-Setting Theory (GST) and Expectancy-Value Theory (EVT) together provide further insight into how learner engagement is initiated and sustained in the language learning process. GST, as articulated by Locke and Latham (1990), emphasizes that clear, challenging, and personally meaningful goals help direct attention, mobilize effort, and maintain persistence over time. In parallel, EVT (Eccles & Wigfield, 2020) explains how learners’ beliefs about their likelihood of success (expectancies) and the value they attach to a task (subjective task value) combine to influence their willingness to engage. In L2 contexts, these dimensions are visible in learners’ confidence and the personal importance they place on achieving language competence (Gaspard et al., 2017; Nagle, 2021).

Within Directed Motivational Currents (DMCs), this connection becomes especially clear: learners often break a larger vision into concrete, manageable subgoals, which not only provide frequent feedback but also reinforce expectancies and sustain perceived task value throughout the process (Henry et al., 2015; Ibrahim, 2016a; Erten & Selcuk, 2017; Gümüş, 2019). Together, GST and EVT thus help explain how clear goal structures and strong success-value beliefs underpin the focused, sustained effort that defines a DMC episode in second language learning.

### 2.4. Empirical Studies

Empirical studies on DMCs have begun to validate and extend these theoretical insights. Early qualitative investigations, such as those conducted by Dörnyei, Ibrahim, & Muir (Dörnyei et al., 2016), revealed that learners who experienced DMCs reported a temporary but profound immersion in their language tasks, accompanied by a noticeable progress in learning outcomes. Ibrahim’s (2016a) phenomenological study further confirmed that DMC launches are marked by distinct, personal triggers, with participants recalling specific moments that initiated their intense motivational states. Quantitative research by Muir (2016) corroborated these findings on a larger scale, demonstrating that a substantial proportion of language learners acknowledge experiencing DMC-type motivation.

Subsequent studies have employed diverse methods—from online questionnaires to in-depth interviews and diary analyses—to investigate DMCs across various cultural and educational contexts. For example, research in Iran (Ghanizadeh & Jahedizadeh, 2017), Turkey (Erten & Selcuk, 2017), and Japan (Yazawa, 2020) has not only validated the existence of DMCs but also examined how factors such as language proficiency and educational settings modulate their occurrence.

Furthermore, several studies have demonstrated that DMCs operate at the group level, underscoring their situational nature as team members influence one another to sustain high engagement in personally and professionally meaningful tasks (García-Pinar, 2022; Zarrinabadi & Khajeh, 2021). Other research has mapped diverse DMC triggers, revealing their context-dependence: negative emotions, emergent opportunities, single explicit goals, inspiring experiences, competition, and social support from teachers or peers all serve as catalysts (Başöz & Gümüş, 2022). Studies of feedback effects show that both positive and negative feedback can, respectively, strengthen or interrupt DMC momentum, influencing learners’ self-efficacy and mood (Zarrinabadi & Soleimani, 2022). Finally, exam preparation has been identified as a fertile ground for DMCs, with learners reporting heightened goal-focus, clear progress perceptions, and perceived behavioral control during intensive exam periods (Shen et al., 2025). Together, these findings illustrate how DMCs emerge from the interplay of multiple factors within a dynamic learning ecology.

More recently, the application of DMC theory has expanded to the Chinese context, where studies have revealed that English learning DMCs are associated with Chinese tertiary students (Li et al., 2021; Chang & Zhou, 2023; Yu & Liu, 2023). Studies also begun to compare DMC experiences among secondary and tertiary English as a Foreign Language (EFL) learners (Huo et al., 2025). Findings indicate that DMC experiences may vary significantly across educational levels, with implications for curriculum design and instructional practices. These studies offer valuable insights into how structured learning environments and peer dynamics influence the formation and sustainability of DMCs.

In summary, while DMCs share commonalities with related motivational constructs, they represent a unique phenomenon characterized by prolonged, goal-directed engagement and positive emotional feedback. The integration of Flow Theory, GST, and EVT provides a comprehensive framework for understanding the triggers, processes, and outcomes associated with DMCs. Nonetheless, further empirical work is necessary to refine theoretical models and enhance measurement tools, particularly in diverse educational and cultural settings. This ongoing research promises to enrich our understanding of motivation in language learning and to inform more effective pedagogical interventions.

In response to the gaps identified above and guided by the research objectives, this study aims to address two major research questions (RQs): How are DMCs triggered? What constitutes a DMC structure? Specifically, we intend to answer the following questions:

(1) What criteria are considered when adopting a DMC structure?
(2) In what ways does a DMC structure sustain motivation throughout its course?
(3) Are there any non-defining features of the DMC framework?

## 3. Methodology

### 3.1. Study Design

This qualitative study is the second phase of an explanatory sequential mixed-methods project investigating DMCs among Chinese EFL learners. The initial quantitative phase (Huo et al., 2025) employed a cross-sectional survey of 740 high school and university students to identify predictors of DMC initiation (e.g., expectancy for success, parental encouragement). To contextualize these findings, this follow-up phase adopted a qualitative dominant design (Creswell & Plano Clark, 2018), combining semi-structured interviews to explore how and why DMCs emerge, evolve, and influence learning outcomes.

The study aligns with Denzin’s (2017) triangulation framework, integrating quantitative and qualitative data to enhance validity. Semi-structured interviews, guided by Ibrahim’s (2016b) DMC protocol (see Appendix A), probed participants lived experiences, focusing on motivational triggers, emotional engagement, and contextual barriers. This approach balanced theoretical grounding (deductive coding) with openness to emergent themes (inductive analysis), ensuring both methodological rigor and exploratory depth (Braun & Clarke, 2006).

### 3.2. Participants

Participants were selected from the pool of respondents in our initial quantitative study based on their scores on the DMC scale. Only those who self-reported having experienced a directed motivational current and whose overall DMC score exceeded 4.0 (out of 5.0) were invited to participate in the follow up interviews. An initial recruitment effort targeting 15–20 such individuals yielded only three volunteers, owing in part to post pandemic research fatigue. To bolster the sample, we then adopted a snowball sampling strategy (Creswell, 2012), asking each of the three engaged participants to refer peers who had completed the original survey and reported experienced DMCs. All referred candidates were invited and interviewed (*N* = 13); from these and the original volunteers, we retained only those whose interview data confirmed a consistently high DMC profile. This procedure ultimately produced a final sample of ten students—five high school and five university—all of whom demonstrated robust, sustained DMC experiences. This strategy captured socioeconomic diversity: urban high school learners (e.g., Qin from affluent backgrounds), small-town students (Hu, middle-class), and rural participants (Yan with resource constraints), alongside university students spanning career-focused International Business majors (Sun) and exam-driven Engineering learners (Dou).

### 3.3. Procedure and Interview Schedule

Data collection employed Ibrahim’s (2016b) DMC protocol through Mandarin semi-structured interviews via the Tencent Meeting platform (similar to Zoom Meeting) (40–60 min each), focusing on three domains: (1) DMC triggers (“Describe a moment you felt unstoppable in learning English”), (2) emotional/cognitive engagement (“How did your motivation evolve?”), and (3) contextual influences (“What barriers/supports shaped your experience?”). To enhance recall accuracy, participants were asked to focus on a recent DMC. Interviews were transcribed verbatim, back-translated for cross-linguistic validity, and member-checked by participants to address ambiguities. All procedures and protocols associated with this study were approved by the University of Auckland’s Human Participants Ethics Committee (protocol number: UAHPEC24419).

### 3.4. Data Analysis

Thematic analysis followed Braun and Clarke’s (2006) six-phase framework, blending deductive coding aligned with Ibrahim’s (2016b) DMC constructs (e.g., goal-orientedness, emotional intensity) and inductive exploration of emergent patterns. The process began with familiarization through iterative transcript reviews to identify recurring patterns, followed by initial coding that applied predefined categories (e.g., “facilitative structures”) while remaining open to novel insights. Theme development grouped codes into overarching themes such as Role Models (e.g., mentions of “role models” (偶像) and “admiration” (敬佩)), with subthemes like Peer Influence vs. Teacher Guidance uncovering nuanced social dynamics. During theme review, internal coherence and external distinctiveness were rigorously evaluated, while refinement integrated unexpected findings (e.g., Rural-Urban Resource Disparities) through iterative recording. Reporting synthesized qualitative insights with quantitative predictors (e.g., parental encouragement) to triangulate DMC mechanisms.

Analytical rigor was strengthened through member checking (participant validation of transcripts) and thick description contextualizing findings within participants’ lived realities, such as Yan’s rural resource constraints, ensuring sociocultural authenticity in interpretations. Also, the analysis balanced theoretical grounding with openness to unanticipated insights. For example, while predefined codes captured expectancy-value dynamics, inductive coding revealed how exam pressures uniquely shaped high school students’ DMCs compared to university learners’ career-driven motivations. This dual approach enriched the explanatory power of findings, bridging quantitative predictors with qualitative depth. This study is the qualitative phase of explanatory sequential mixed methods research. As detailed below, this design integrates the use of quantitative and qualitative approaches and allows for the triangulation of distinct data sources, surveys and semi-structured interviews in this case. Triangulation, as further clarified by Denzin (2017), involved the use of multiple methodologies to examine the same phenomenon, with the goal of gaining a comprehensive understanding. The deployment of various methods in this research was intended to ensure a deep insight into the topic being investigated.

## 4. Findings and Discussion

### 4.1. Mapping DMC Progression from Trigger to Trajectory

**Figure 1:**
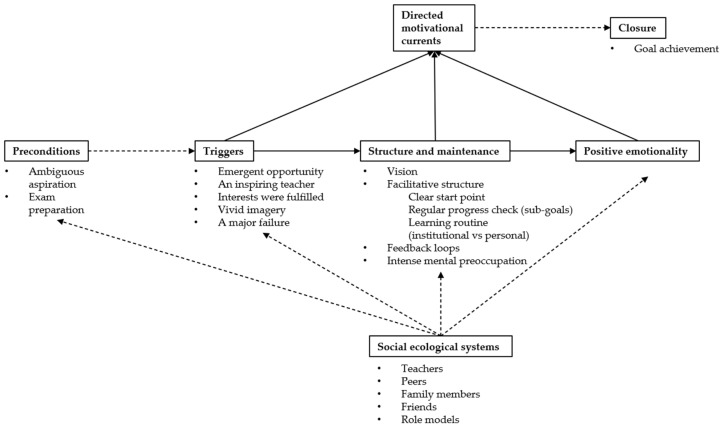
Ecological DMC model.

As depicted in Figure 1, all participants’ experiences exhibited the hallmark DMC structure: a clear trigger, sustained high energy through strategic sub-goals, and a natural wind-down upon goal completion. As one learner put it, they moved from an initial spark—“envisioning admission to a top university or study abroad”—to weeks or months of disciplined routines and progress checks, and finally to a sense of closure when the exams were passed. This closed-loop pattern aligns precisely with Dörnyei et al.’s (2015) DMC model.

Informed by the thematic analysis, the results are organized into themes and subthemes addressing the overarching research inquiries. These interviews, coupled with the survey study (Huo et al., 2025), served to triangulate the findings from the quantitative research. Due to strict word-count limits, we abbreviated most examples from the dataset; However, we selectively incorporated longer textual excerpts whenever necessary to preserve discourse coherence.

Furthermore, in the preliminary phase, participants explicitly articulated their strategic approaches to language acquisition, with those available for subsequent interviews providing deeper insights. Through iterative phenomenological coding, we identified 14 distinct thematic clusters representing recurrent patterns across the dataset. These analytical constructs systematically map onto the core dimensions of the DMC framework, elucidating key correlates of its structural architecture. The following synthesis organizes these findings according to the six-phase DMC developmental model, maintaining constant dialogic alignment with the study’s research questions.

Regarding the circumstances prior to and around the time of the DMCs, the analysis identified four major themes (e.g., the DMC launch, a DMC structure) and several sub-themes (e.g., clear starting point, the DMC triggers).

### 4.2. Theme 1: The DMC Launch

#### 4.2.1. Pre-Conditions for DMC

The mere presence of a triggering event alone was insufficient to initiate a DMC; activation also required specific preconditions—whether internal (e.g., a goal-oriented mindset) or external. In this study, we found that most participants already held a future vision prior to entering a DMC. Although a few had a special interest in improving their English, their initial goals were generally centered on broad self-improvement rather than direct language acquisition. Consequently, these pre-trigger goals represented general aspirations for a better future.

Table 1 summarizes participants’ pre-trigger goals. Only Kiki and Du explicitly aimed to achieve a certain level of L2 proficiency; the others viewed proficiency as a means to attain their broader objectives. Upon encountering a triggering stimulus, participants transitioned to a post-trigger state in which their objectives became specific: ambiguous aspirations evolved into clear, actionable goals directly linked to L2 learning. This shift—from general vision to concrete language-related targets—was essential for directing sustained effort toward a well-defined endpoint.

#### 4.2.2. Clear Starting Point and DMC Triggers

All ten participants vividly remembered the start of their DMC experiences. They not only anchored the beginnings of their motivational journeys to specific events or significant moments, but they also provided comprehensive details about the circumstances preceding and surrounding the onset of their experiences. For example, in describing her DMC related to pursuing, Yan stated, “From the very first class at my high school, my teacher portrayed university life as exceptionally beautiful.” Similarly, Hu could not forget his failure in the university entrance examination and the DMC it triggered: “On the first day of my fourth year of high school, I realized that it was my last chance to get into university.” And Chen could even recall the exact date of the onset of DMC experience: “On that day, I knew my school would select students to form a special class for the preparation to enter the key high school.”

These data delineate how specific contextual triggers spontaneously elicited profound emotional surge among participants. The combination of task complexity, challenge, and perceived value strengthened their willingness to take on demanding, goal-oriented work. These findings illustrate how DMCs typically begin and confirm that such contextual triggers serve as catalysts during their stage of a DMC.

#### 4.2.3. The DMC Triggers

In addition to identifying a clear starting point, each participant detailed the catalyst for their DMC initiation. The outset of their experiences was variably linked to or caused by a unique incident or as a result of a specific triggering stimulus. All participants had experienced one or more of the following initiating triggers.

**Emergent opportunity.** During the last year of junior high school, Chen’s school announced that there would be a special class choosing the top 10 students from each class aiming at preparing them for direct admission for the best local high school. Chen’s aspiration to be selected for this program, thereby significantly increasing his chances of gaining entry into one of the best local high schools, acted as the trigger for his DMC:

When I heard this program, my ambition sparked. To be one of this elite group and to attend the best high school became a fervent desire within me. Consequently, I started to take English study seriously which I previously overlooked.

**Meeting teachers who were inspiring.** Qin and Yan described how their new teachers influenced them to be interested in English learning. Either the unique way of English teaching or the talent of portraying a promising future for students, arousing participants’ interests in English learning and dedicated their effort on the subject. As Qin stated:

When I entered junior high school, my new English teacher organized the classes in a manner that captivated our interest, designing a variety of activities that encouraged interaction both between teacher and students, and among the students themselves, which let him create an immersive English-speaking environment, compelling us to communicate in English…

Similarly to Qin’s teacher, Yan’s teacher was good at painting vivid pictures of a promising future, consistently captivating students with visions of what lay ahead:

…my English teacher often told stories of her university life which was vivid and captivated…

**Interests were fulfilled**. Kiki and Chen reported that their long-standing interest in learning English was revitalized through the introduction of new approaches or opportunities for further developed. This infusion of fresh approaches re-energized their English learning pursuits, leading them emersed themselves in the new learning activities immediately. Kiki felt lucky that she received an offer with her dream university and favorite major. She experienced a sense of beginning anew, as if a new door had opened to her:

I was so happy to major in interpretation and international trade. Upon my first arrival on campus, everything felt fresh and exhilarating. Motivated by this new beginning, I committed to diligently studying English, aiming to secure a position at international companies in the future.

**Vivid imagery.** Four participants mentioned envisioning their moment of success, each crafting a detailed and vivid depiction of their future lives in their minds. For instance, while Yan’s teacher provided an engaging introduction to high school and painted an attractive image of university life, Yan herself conjured an even more vivid and detailed mental image. Her vision encompassed scenes of campus landscapes, studying in the library, participating in extracurricular activities, and experiencing romantic relationships on campus. These images drove her to study more diligently.

**Stimulated by a major failure.** The data revealed that failure had triggered the participants who possessed an exceptionally strong sense of self-esteem to start a DMC. Two participants exhibited their determination after they failed the university entrance examination. As Hu stated:

Both my family and I were deeply disappointed by my result. I never anticipated that I was rejected by all the universities that I had applied. Particularly, when I saw most of my classmates enrolled in the universities, I felt an intensified sense of shame, anger, fear and resentment towards myself. However, I was not ready to concede to defeat. I swore to study hard, determined to reattempt the exam next year. I became so focused on my preparations that it felt as if I had isolated myself from the outside world, dedicating countless days and nights to studying in the classroom, avoiding all kinds of entertainment.

Ya also performed poorly in the exam. However, she strategically rechanneled this setback into a motivational catalyst, formulating a structured goal-orientation plan for IELTS preparation and for international graduate study. This behavioral pattern aligned with Muir’s (2016, p. 44) conceptualization of negative affect triggers, wherein ‘experiences of academic embarrassment, perceived humiliation, or goal-attainment failures’ possessed triggering capacity for motivational restructuring. This study revealed that participants successfully transformed such affective challenges into strategic pivots can then trigger DMCs.

Furthermore, these triggers underscore how DMCs align with GST and EVT. When participants set clear, challenging goals—such as gaining entry to a prestigious university or studying abroad—they placed high value on every preparatory task. By reflecting on past failures and comparing themselves to similarly situated peers who succeeded, they strengthened their expectancy of success. This combination of specific, demanding goals and strong task value and expectancy beliefs fueled the prolonged, focused effort that defined their sustained motivational currents.

### 4.3. Theme 2: Developing a DMC Structure

#### 4.3.1. Invest in Extra Time

In all the DMC cases, participants perceived a heightened sense of time scarcity following the initiation of a DMC. This realization spurred them into dedicating more time and energy towards their endeavors, aiming to transform their passion into tangible actions. Chen learned about the selection for a special class and he instinctively knew that an increased investment of time in his studies was imperative:

… I first realized that I needed to devote more time and effort to my English study. I used to take naps at noon, but I quit it and would head straight back to classroom after lunch. Moreover, I extended my study hours until midnight.

Chen recalled being uncertain about how to achieve his goal at the beginning. He was driven by a sense of urgency, understanding that dedicating more time and being more engaged in his English studies would increase his chances of discovering an effective and productive approach to enhance his learning.

#### 4.3.2. Urge to Develop a DMC Structure

After investing more time in their motivated behaviors, all participants experienced an urgent need to establish a structured approach that would allow them to focus better and be more effective and productive. Yan reflected on this, noting:

…though I managed to adhere to the schedule aligned with our monthly exams, I realized it was insufficient. I envisioned a new schedule in my mind, one that could accommodate my learning needs, amplify my strengths and addressing my weaknesses.

Her expression had clearly revealed that she had developed a clear goal/vision and simultaneously tried to devise a strategy to realize that vision.

#### 4.3.3. Institutionalized Structure vs. New Learning Structure

Initially, all participants might rely on previous learning methods or school designed structure to embark on this journey. However, they quickly recognized their limitations and swiftly adapted, creating a new structure for their studies. Consequently, even in the absence of the best pre-planned structure or method, these participants appeared to utilize the resources and opportunities immediately available to them.

Hu was an example of the high school participants. His school offered a thorough curriculum for students aiming for the university entrance exams. Yet, he noted the English classes were heavily grammar-focused and exam-oriented. While recognizing their importance, Hu realized he required additional supplementary learning:

… school classes were important, yet the memorization techniques my teacher employed were not effective for me. The best way for me to retain vocabulary was to incorporate these words into my daily life. Typically, the English teacher would highlight key words on the blackboard, and I would select those I found challenging. Lacking an English-speaking environment, I resorted to crafting stories or dialogues and engaging in solo role-play.

It is crucial to note that nearly all participants initiated their journey by adopting behavioral changes to align with their valued goals. Despite entering the DMCs without ample preparation, they swiftly adapted, creating a new structure for their studies.

#### 4.3.4. Criteria Used in Selecting a DMC Structure

Subsequent to the determination of how participants established their DMC structures, it becomes essential to explore the criteria informing them of their choices. In Ibrahim’s (2016b) study, he identified two primary criteria for selecting a DMC structure: personal appropriateness and goal appropriateness, both of which align with Locke and Latham’s (1990) notion of a personally valuable goal. Our findings resonate with their work. However, a notable divergence was that the participants in this study, instead of rejecting others’ advice, displayed openness.

### 4.4. Theme 3: DMC Structure and Maintaining Motivation

Five key themes were identified in this study, except for *Adjustment of a DMC routine*, other four themes were aligned with the previous studies (Dörnyei et al., 2015; Safdari & Maftoo, 2017; Zarrinabadi & Tavakoli, 2017; Oh & Kim, 2024).

#### 4.4.1. Developing Behavioral Routines

Almost all the participants reported a rapid increase in time consuming on their study, quickly establishing a new learning routine. They vividly remembered their daily activities, highlighting the presence of behavioral routines as a prominent theme. This entailed relying on a nearly systematic set of recurring learning behaviors.

Hu as well as other high school students in China were part of a tightly structured academic environment, with classes meticulously designed by schools to enhance their exam performance. Despite these constraints, they crafted personal routines around the school’s timetable to meet their DMC objectives:

I attended one or two English classes daily, where our teacher focused on exam preparation … my own routine went like: I woke up 30 min early every morning to listen to English news or dialogues…

University students, on the other hand, experienced greater autonomy in managing their time. Experiencing a DMC immediately upon entering university can greatly ease the transition from high school to tertiary education. As Kiki reflected on her English learning upon entering university:

…Upon entering university, my schedule was no longer as tight. My focus shifted solely to improving my English. I resorted to using task lists and timer apps on my phone to keep track of the goals I set for myself. Completing these tasks daily brought me immense pleasure and a profound sense of satisfaction.

However, this is not a natural process. Students face the challenge of high self-discipline, and not every college student can swiftly adjust their study routines to the new learning environment. As Kiki noted, while she thrived in her English studies, some of her peers struggled to find their footing in the highly flexible university life.

#### 4.4.2. Adjustment of a DMC Routine

The establishment of a new learning routine did not signify permanence; participants reported continually tweaking these routines as they acquired new learning skills or resources. Even minor adjustments could significantly impact their DMC experiences, highlighting the dynamic nature of their learning journey. Kiki elaborated:

During a simulated international business interpretation assignment, I learned to use PowerPoint to showcase our products. This opportunity was a novel experience for me. It was exhilarating to engage with learning material outside of textbooks. From that point on, I created and shared several slides with my peers every week.

Using digital technique and learning new skills were incredible experiences for university students, which renewed their learning methods, increased their sense of ownership, and engagement in English learning.

#### 4.4.3. Intense Mental/Affective Preoccupation

Despite the establishment of behavioral routines, deep engagement with the DMC process was prevalent among participants. Analytical findings revealed that most learners employed multifaceted strategies rather than isolated activities, integrating diverse resources such as lexical curation (e.g., collecting idiomatic expressions), structured coursework (e.g., attending classes, completing assignments), immersive input (e.g., listening/reading authentic materials), metacognitive practices (e.g., error notebook reviews, Feynman Technique application), and creative output (e.g., crafting dialogs, solo role-play, oral presentation). While daily time investment varied (1–6 h), participants consistently prioritized sustained, goal-aligned engagement over fragmented study. Moreover, although participants reported numerous flow states during these individual tasks, these brief episodes invariably subsided once the task was completed. In contrast, their DMCs endured for months—or even up to a year—providing the sustained momentum needed to overcome recurring challenges and ultimately achieve their overarching goals.

#### 4.4.4. Manageable Subgoals

Upon entering a DMC experience, most participants segmented their ultimate goal into manageable short-term tasks, even without clear foresight of each sub-goal’s attainability. As their journey progressed, they gained a nuanced understanding of their English proficiency, prompting adjustments to both their sub-goals and overall strategies. Based on ongoing external and self-assessment, some accelerated their efforts with more ambitious plans, while others decelerated to realign their objectives with evolving circumstances. Hu set monthly objectives based on the structure of the school curriculum, targeting an improvement in the ranking positions each month. As Hu observed progress in the initial months, he increased his study intensity and experimented with new learning techniques:

… I began incorporating additional vocabulary from news sources into my word list and engaged in solo role-play exercises to practice these new words and phrases.

University participants appeared to enjoy greater flexibility in organizing their studies. However, this flexibility often introduced additional challenges due to the absence of a specific structure for monitoring progress. Ya shared her strategies for learning English:

…Then, I adopted a two-step strategy. Initially, I enrolled in an off-campus institution’s summer camp focused on IELTS exam preparation. The second phase involved crafting my individual plan, incorporating diverse learning resources from both the institution and online platforms. I set specific subgoals, such as passing the CET4 and CET6 exams, and devised detailed plans targeting vocabulary, grammar, speaking, listening, and reading skills.

In summary, both groups concluded that various exams served as the primary foundation for formulating their learning plans and assessing their capabilities. They highlighted the significance of setting manageable subgoals, which maintained their English DMCs.

#### 4.4.5. Feedback Loops

In the previous sections, participants had mentioned the feedback they received helped them keep on DMC experiences. High school participants received feedback regularly from their teachers due to formal courses and monthly exams. Qin recounted how her teacher’s varied feedback consistently brought her pleasant surprises and became the highlight of her day:

I eagerly anticipated my teacher’s feedback on each of my assignments or exam papers. Whether it was corrections of my mistakes or praise for my progress, both were vital to me, providing benchmarks to evaluate my improvement. Additionally, whenever I raised my hand and answered questions in English in front of my class, I looked forward to my teacher’s commendation, or even just a smile or nod, as encouragement.

University participants, in contrast, faced a scarcity of institutionalized feedback, prompting them to source feedback from a variety of channels. Dou, for instance, crafted essays on IELTS topics and eagerly presented them to his teacher for critique. Ya, aiming to enhance her speaking and writing abilities, engaged an online one-on-one IELTS tutor for practice sessions, receiving detailed and professional evaluations of her English skills. Kiki, intentionally seeking feedback, turned to her peers for their insights. All forms of objective or positive feedback served as the motivation for their continued efforts and engagement in English learning.

### 4.5. Theme 4: DMC Closure

This section comes to the discussion of the final stage of the DMC experiences, aiming at exploring the end point of a DMC experience and finding out whether a DMC experience helped participants achieve their final goals and whether the structure continued to be useful for following DMC experiences.

#### Patterns of DMC Closure

All participants noted a specific end point of their DMC, which typically coincided with the completion of their exams. Of the nine who achieved their initial DMC goals, two participants had not undergone a second DMC. Yan and Qin found that the strategies which served them well previously did not translate effectively to their new DMC experiences. Yan reflected on this disparity:

The methods that worked for my university entrance examination proved ineffective for my current university studies…the nature of the challenges I now face has changed. University life is more flexible, presenting numerous distractions. The lack of external pressure and clear direction is notable… In the absence of exams, I struggle to gauge my English proficiency, particularly my speaking skills.

Moreover, four participants had a second DMC experience and achieved success again. Kiki was the first to acknowledge the distinct environment of university life early on. Upon her university entrance, she set a clear objective to become an interpreter and engage in international business, adapting elements of her prior strategy accordingly:

I am aware that university is full of distractions, making it challenging to adhere strictly to the methods I used in high school. While I continue to attend classes and visit the library daily, I no longer depend solely on my teachers or focus my efforts around exam preparation.

Additionally, Kiki embraced a wide array of learning opportunities, integrating various new elements into her updated DMC experience, such as online resources, collaborating with other students, organizing English activities.

In summary, it is clear that a DMC experience usually concludes upon the achievement of its set goal. The knowledge and insights gained from a previous DMC can undoubtedly assist in initiating and sustaining a new DMC. However, this does not guarantee that the strategies and structures from a past DMC will be entirely applicable in a new context. Therefore, it is crucial for students to thoroughly understand and adapt to their evolving situations. By contrast, general long-term motivation in L2 learning typically lacks such discrete triggers and milestones, instead meandering around broad aspirations (e.g., “I want to be fluent one day”) without a defined endpoint. Flow experiences, though intensely immersive, are inherently transient—lasting mere minutes or hours during a single task (Csikszentmihalyi, 1988). Learners themselves drew the distinction, contrasting flow’s fleeting “losing track of time” moments with the sustained “marathon of motivation” that defined their DMC journeys.

## 5. Conclusions

Previously published quantitative research demonstrated that DMCs are widely recognized and influential among adolescent Chinese EFL learners, linking them to personal salience, expectancy for success, and parental encouragement, with improvements in self-assessed English proficiency underscoring their practical significance in L2 teaching (Huo et al., 2025). This second-phase qualitative study, which triangulated the mixed-methods design, provides robust evidence by identifying a DMC structure composed of both school-generated and self-generated elements—such as a clear starting point, established routines, affirmative feedback, regular progress checks, and subgoals—that sustain these motivational currents. Participants who took personal ownership, driven by a clear vision of their ideal academic selves, benefitted from supportive teachers and a nurturing classroom environment that quickly established the necessary motivational structures. In addition positive emotionality—stemming from goal-oriented progress, eudaimonic happiness, and familial expectations—fostered excitement, confidence, and resilience, enabling them to overcome challenges and achieve stronger learning outcomes.

The implications of the study’s findings highlight distinct yet interconnected strategies for high school learners, university students, and educators. For high school learners, diversifying learning resources (e.g., online materials, peer collaborations) beyond textbooks and integrating school curricula with personalized plans can enhance productivity, while interactive English activities (e.g., clubs, competitions) alleviate exam stress and foster peer-driven motivation through shared goals. University students, meanwhile, should prioritize autonomous learning by aligning studies with personal interests and career visions, designing self-directed activities (e.g., language cafes, project-based tasks) to complement formal education and transition from teacher reliance to proactive skill-building. Moreover, educators could play a critical role in bridging these stages. Although many students emphasized their personal agency in choosing learning methods, well-planned pedagogical support remains crucial, especially for high school learners with less developed autonomy. Teachers can trigger and sustain motivation by designing clear pathways, helping students envision outcomes, and adding flexible, student-driven tasks. Drawing on the ecological nature of DMCs, integrating student-led practices and family support can also build an agency, reduce anxiety, and strengthen collective motivation, especially for those navigating intense exam pressures.

The current study has several limitations that offer avenues for future research. First, our cross-sectional, retrospective design cannot fully capture the dynamic nature of motivation and is susceptible to recall bias. Moreover, this study only touched briefly on potential side effects of DMCs—particularly negative emotions arising from high-pressure exams. Although none of our participants explicitly reported breaks or setbacks in their DMCs due to anxiety or stress, we cannot assume these issues were absent. Future research should investigate the extent to which exam-related pressure and other negative emotions may disrupt, diminish, or otherwise shape learners’ DMC experiences. To address these limitations, future research should adopt longitudinal observational and experimental designs to establish causal relationships and control for confounding variables over time. Additionally, micro-genetic approaches are recommended to capture the nuanced, day-to-day fluctuations in learning motivation both in and out of classrooms. Such designs would yield a more comprehensive understanding of the multifaceted dynamics of DMC experiences, refining theoretical constructs and informing effective pedagogic strategies.

Finally, this study advances research on DMCs among Chinese adolescent EFL learners, addressing gaps in understanding sustained motivation dynamics. Theoretically, it refines DMC theory by identifying essential preconditions (e.g., supportive environments, prior inspiration) that must precede external stimuli to trigger motivation—highlighting the insufficiency of isolated triggers. By contextualizing DMCs within adolescent learners and contrasting high school and university cohorts, it expands SLA research into non-Western settings, revealing how developmental stages shape directed motivational trajectory. Methodologically, this triangulation offers a replicable framework for exploring complex motivational phenomena, while practically, it provides actionable strategies for educators to cultivate sustainable language learning motivation, thereby informing both theory and practice in second language acquisition.

## Figures and Tables

**Table 1:** Participants’ Goals/ambitions.

Participant	Pre-Trigger Goal	Post-Trigger Goal
1. Chen	To enter a high school and study in a competitive environment.	To gain high scores in English and enter a target high school.
2. Yan	To gain an opportunity to enroll in a prestigious university.	To gain high scores in English which is his weak point.
3. Sun	To further her study overseas, broaden her horizon and prepare her for the future competitive work market.	To improve her English and pass IELTS examination.
4. Qin	No specific goal was mentioned.	To improve her English proficiency and gain high scores.
5. Hu	To catch up with others and enter a prestigious university.	To gain high scores in English which is his weak point.
6. Dou	To secure a bright future life and prove himself to relatives.	To gain high scores in English and pass exam.
7. Wang	To enter best high school and best university, to be an elite in the society.	To seek every source benefiting his math and English advancement.
8. Kiki	To be an interpreter and to do international business.	To study English diligently on interpretation.
9. Du	To enhance her English skills, enabling her to live freely in a foreign country.	Stick to the plan arranged by App to keep learning English.
10. Ya	To gain admission to top-tier universities for her graduate study.	To improve her English and pass IELTS examination.

## Data Availability

Data will be available upon reasonable request.

## Appendix A. Individual DMCs Interview Procedures and Protocol

As a second language learner, can you reflect back and think about your experience. Did you have a period of your life when you were extensively involved in learning and improving your English to reach a high value goal such as a trip to an English-speaking country, improving your English for job-related goals, or preparing to study in an English-speaking country, or any other personal goal. Do you remember this period of time as one in which you were highly motivated, passionate and enthusiastic because you were experiencing something a bit unusual in regard to your interest to reach a high degree of proficiency?

To make things clearer, consider this example: Someone decides to start learning a foreign language in preparation for an extended foreign trip, and becomes embroiled in the process to such an extent that she spends virtually all her free time studying the language, while also purchasing dictionaries and computer software to direct her learning as well as voraciously reading guidebooks and surfing English websites to familiarize herself with the English culture and environment. In an extreme case, she might bore family and friends rigid by talking of the trip and the language incessantly, may dream of the journey at night and cannot help but rehearse the language even while lying in bed. It is as if a new world had opened up for her and, up until the journey, her pursuit of this newly found vision becomes one of the most significant parts of her life.

If a similar circumstance happened to you, can you please think and reflect on the following areas:

- What made this dramatic change to happen? The main reasons and factors that made this get started. Discuss the circumstances that led to that journey.
- What was your goal?
- How was your motivation during this period toward learning L2?
- Was your motivation qualitatively and/or quantitatively different during this period in comparison to before and after?
- Did you feel something different going on in your life during that period? Why?
- When you reflect back on that experience, what comes to your mind and what do you miss about it?
- Did you achieve your goal? How long did it continue?
- What was your feeling during that period?

More specific questions on goal-orientedness in DMC:

- Was your goal personally important? Why?
- Did you also envision receiving your goal (imagining the moment of getting your goal)?
- In addition to your final goal, how did you set your immediate or shorter-term goals?

More specific questions on Salient Structure in DMC:

- What plan/mechanisms/resources did you use? Did doing this become a daily routine?
- Discuss your daily life in regard to your engagement in improving your language during this time?
- Did you take a course or was it based on a personalized learning plan?
- How did you measure your progress?
- Did you attempt to receive guidance from others, for example from a teacher or a more competent peer?
- Did you break your goals into short and long-term goals? How? Why?
- How long were you studying during this time? Discuss your daily engagement with L2 learning.
- How long did it last for?

More specific questions on Positive Emotional Loading in DMC:

- Did you feel happy during that period? Why?
- Did you feel excited? What was it that you were excited about?
  - Did you not try to share your excitement and happiness with other people? Why/Why not?
- How was the quality of time spent on learning/improving language in that experience?
- Did you enjoy effort? Or was it felt like a burden? Did you even enjoy the difficulties? Why?
  - Did this experience bring meaning to your life at that time? Why?
- Did other people tell you or think you were making a big deal of this experience?
  - In what way did this engagement develop your potential?
- In what way, did it make you a better, more skillful person?
  - During that period, how important this had become in your life? Was it important or not so important?
- How being successful in this experience give you the sense of discovering your potential?
- Were you concerned/upset that this period and experience was finally over?
- Have you tried to repeat the same experience? Have you been successful? Why/Why not?
